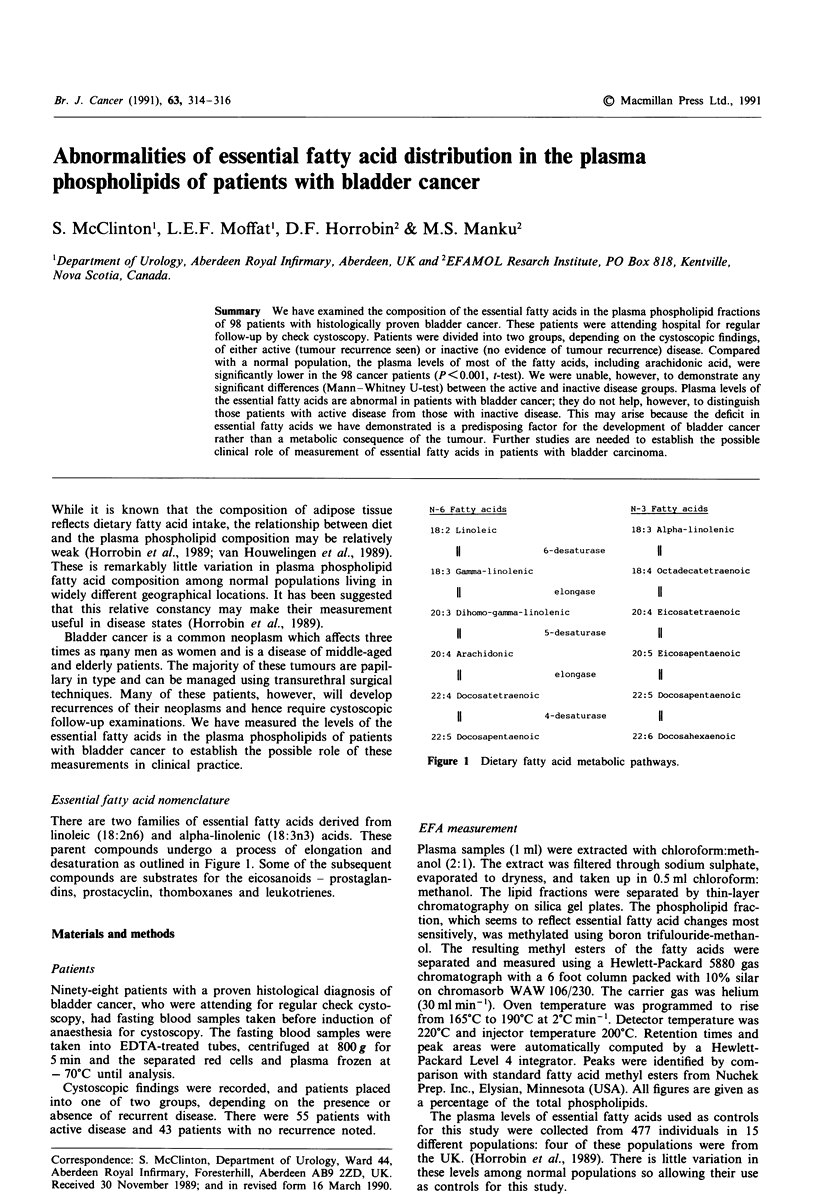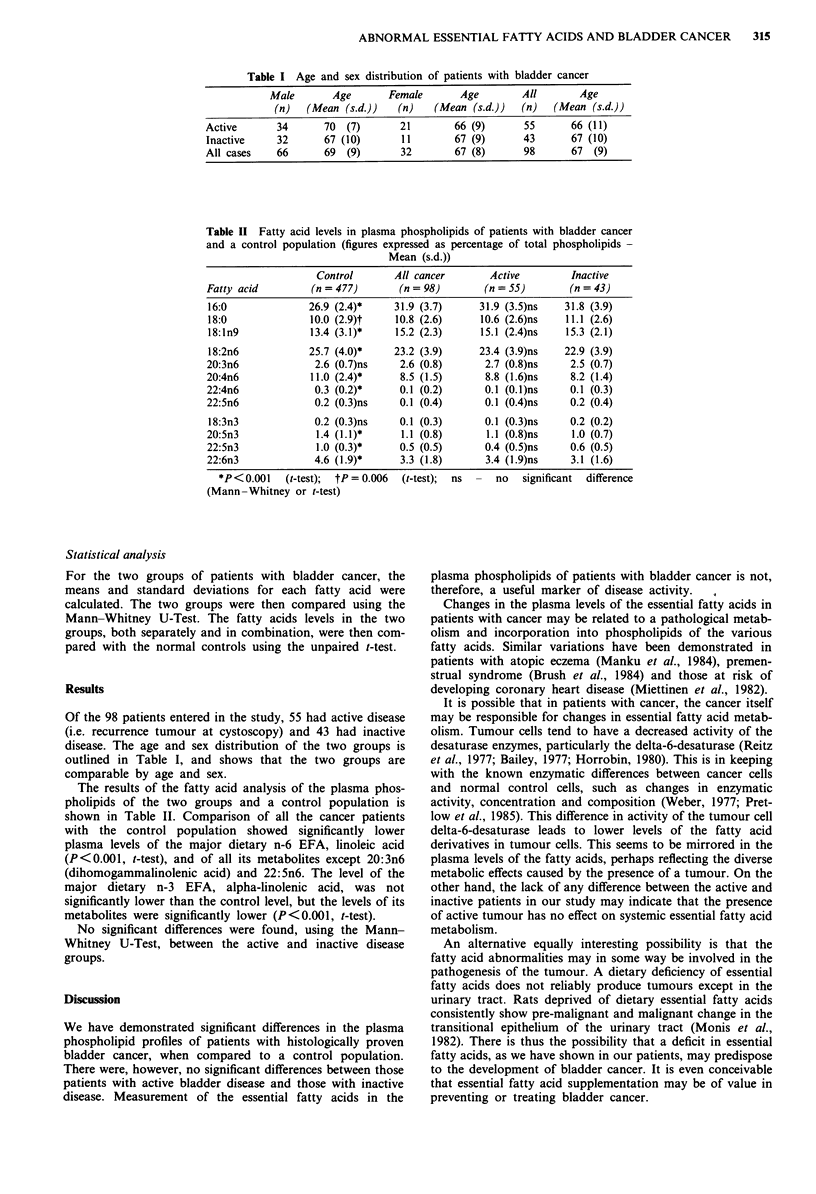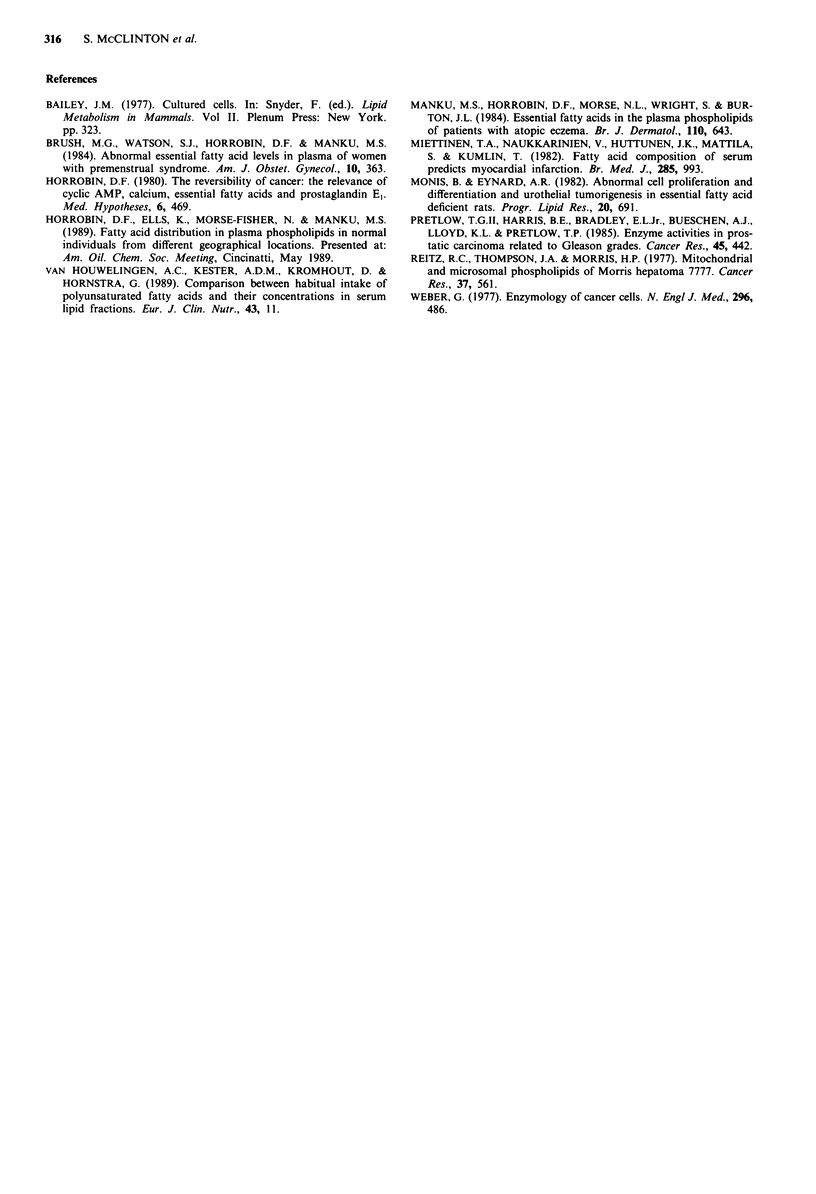# Abnormalities of essential fatty acid distribution in the plasma phospholipids of patients with bladder cancer.

**DOI:** 10.1038/bjc.1991.73

**Published:** 1991-02

**Authors:** S. McClinton, L. E. Moffat, D. F. Horrobin, M. S. Manku

**Affiliations:** Department of Urology, Aberdeen Royal Infirmary, UK.

## Abstract

We have examined the composition of the essential fatty acids in the plasma phospholipid fractions of 98 patients with histologically proven bladder cancer. These patients were attending hospital for regular follow-up by check cystoscopy. Patients were divided into two groups, depending on the cystoscopic findings, of either active (tumour recurrence seen) or inactive (no evidence of tumour recurrence) disease. Compared with a normal population, the plasma levels of most of the fatty acids, including arachidonic acid, were significantly lower in the 98 cancer patients (P less than 0.001, t-test). We were unable, however, to demonstrate any significant differences (Mann-Whitney U-test) between the active and inactive disease groups. Plasma levels of the essential fatty acids are abnormal in patients with bladder cancer; they do not help, however, to distinguish those patients with active disease from those with inactive disease. This may arise because the deficit in essential fatty acids we have demonstrated is a predisposing factor for the development of bladder cancer rather than a metabolic consequence of the tumour. Further studies are needed to establish the possible clinical role of measurement of essential fatty acids in patients with bladder carcinoma.


*Br. J. Cancer* (1991), **63**, 314–316 © Macmillan Press Ltd., 1991

### Abnormalities of essential fatty acid distribution in the plasma phospholipids of patients with bladder cancer

S. McClinton[1], L.E.F. Moffat[1], D.F. Horrobin[2] & M.S. Manku[2]

[1]*Department of Urology, Aberdeen Royal Infirmary, Aberdeen, UK and* [2]*EFAMOL Resarch Institute, PO Box 818, Kentville, Nova Scotia, Canada.*

**Summary** We have examined the composition of the essential fatty acids in the plasma phospholipid fractions of 98 patients with histologically proven bladder cancer. These patients were attending hospital for regular follow-up by check cystoscopy. Patients were divided into two groups, depending on the cystoscopic findings, of either active (tumour recurrence seen) or inactive (no evidence of tumour recurrence) disease. Compared with a normal population, the plasma levels of most of the fatty acids, including arachidonic acid, were significantly lower in the 98 cancer patients ($P < 0.001$, $t$-test). We were unable, however, to demonstrate any significant differences (Mann–Whitney U-test) between the active and inactive disease groups. Plasma levels of the essential fatty acids are abnormal in patients with bladder cancer; they do not help, however, to distinguish those patients with active disease from those with inactive disease. This may arise because the deficit in essential fatty acids we have demonstrated is a predisposing factor for the development of bladder cancer rather than a metabolic consequence of the tumour. Further studies are needed to establish the possible clinical role of measurement of essential fatty acids in patients with bladder carcinoma.

While it is known that the composition of adipose tissue reflects dietary fatty acid intake, the relationship between diet and the plasma phospholipid composition may be relatively weak (Horrobin *et al.*, 1989; van Houwelingen *et al.*, 1989). These is remarkably little variation in plasma phospholipid fatty acid composition among normal populations living in widely different geographical locations. It has been suggested that this relative constancy may make their measurement useful in disease states (Horrobin *et al.*, 1989).

Bladder cancer is a common neoplasm which affects three times as many men as women and is a disease of middle-aged and elderly patients. The majority of these tumours are papillary in type and can be managed using transurethral surgical techniques. Many of these patients, however, will develop recurrences of their neoplasms and hence require cystoscopic follow-up examinations. We have measured the levels of the essential fatty acids in the plasma phospholipids of patients with bladder cancer to establish the possible role of these measurements in clinical practice.

| N-6 Fatty acids | | N-3 Fatty acids |
|---|---|---|
| 18:2 Linoleic | | 18:3 Alpha-linolenic |
| ‖ | 6-desaturase | ‖ |
| 18:3 Gamma-linolenic | | 18:4 Octadecatetraenoic |
| ‖ | elongase | ‖ |
| 20:3 Dihomo-gamma-linolenic | | 20:4 Eicosatetraenoic |
| ‖ | 5-desaturase | ‖ |
| 20:4 Arachidonic | | 20:5 Eicosapentaenoic |
| ‖ | elongase | ‖ |
| 22:4 Docosatetraenoic | | 22:5 Docosapentaenoic |
| ‖ | 4-desaturase | ‖ |
| 22:5 Docosapentaenoic | | 22:6 Docosahexaenoic |

**Figure 1** Dietary fatty acid metabolic pathways.

### *Essential fatty acid nomenclature*

There are two families of essential fatty acids derived from linoleic (18:2n6) and alpha-linolenic (18:3n3) acids. These parent compounds undergo a process of elongation and desaturation as outlined in Figure 1. Some of the subsequent compounds are substrates for the eicosanoids – prostaglandins, prostacyclin, thomboxanes and leukotrienes.

### Materials and methods

### *Patients*

Ninety-eight patients with a proven histological diagnosis of bladder cancer, who were attending for regular check cystoscopy, had fasting blood samples taken before induction of anaesthesia for cystoscopy. The fasting blood samples were taken into EDTA-treated tubes, centrifuged at 800 *g* for 5 min and the separated red cells and plasma frozen at – 70°C until analysis.

Cystoscopic findings were recorded, and patients placed into one of two groups, depending on the presence or absence of recurrent disease. There were 55 patients with active disease and 43 patients with no recurrence noted.

Correspondence: S. McClinton, Department of Urology, Ward 44, Aberdeen Royal Infirmary, Foresterhill, Aberdeen AB9 2ZD, UK.
Received 30 November 1989; and in revised form 16 March 1990.

### *EFA measurement*

Plasma samples (1 ml) were extracted with chloroform:methanol (2:1). The extract was filtered through sodium sulphate, evaporated to dryness, and taken up in 0.5 ml chloroform: methanol. The lipid fractions were separated by thin-layer chromatography on silica gel plates. The phospholipid fraction, which seems to reflect essential fatty acid changes most sensitively, was methylated using boron trifulouride-methanol. The resulting methyl esters of the fatty acids were separated and measured using a Hewlett-Packard 5880 gas chromatograph with a 6 foot column packed with 10% silar on chromasorb WAW 106/230. The carrier gas was helium (30 ml min$^{-1}$). Oven temperature was programmed to rise from 165°C to 190°C at 2°C min$^{-1}$. Detector temperature was 220°C and injector temperature 200°C. Retention times and peak areas were automatically computed by a Hewlett-Packard Level 4 integrator. Peaks were identified by comparison with standard fatty acid methyl esters from Nuchek Prep. Inc., Elysian, Minnesota (USA). All figures are given as a percentage of the total phospholipids.

The plasma levels of essential fatty acids used as controls for this study were collected from 477 individuals in 15 different populations: four of these populations were from the UK. (Horrobin *et al.*, 1989). There is little variation in these levels among normal populations so allowing their use as controls for this study.

**Table I** Age and sex distribution of patients with bladder cancer

| | *Male (n)* | *Age (Mean (s.d.))* | *Female (n)* | *Age (Mean (s.d.))* | *All (n)* | *Age (Mean (s.d.))* |
|---|---|---|---|---|---|---|
| Active | 34 | 70 (7) | 21 | 66 (9) | 55 | 66 (11) |
| Inactive | 32 | 67 (10) | 11 | 67 (9) | 43 | 67 (10) |
| All cases | 66 | 69 (9) | 32 | 67 (8) | 98 | 67 (9) |

**Table II** Fatty acid levels in plasma phospholipids of patients with bladder cancer and a control population (figures expressed as percentage of total phospholipids – Mean (s.d.))

| *Fatty acid* | *Control (n = 477)* | *All cancer (n = 98)* | *Active (n = 55)* | *Inactive (n = 43)* |
|---|---|---|---|---|
| 16:0 | 26.9 (2.4)* | 31.9 (3.7) | 31.9 (3.5)ns | 31.8 (3.9) |
| 18:0 | 10.0 (2.9)† | 10.8 (2.6) | 10.6 (2.6)ns | 11.1 (2.6) |
| 18:1n9 | 13.4 (3.1)* | 15.2 (2.3) | 15.1 (2.4)ns | 15.3 (2.1) |
| 18:2n6 | 25.7 (4.0)* | 23.2 (3.9) | 23.4 (3.9)ns | 22.9 (3.9) |
| 20:3n6 | 2.6 (0.7)ns | 2.6 (0.8) | 2.7 (0.8)ns | 2.5 (0.7) |
| 20:4n6 | 11.0 (2.4)* | 8.5 (1.5) | 8.8 (1.6)ns | 8.2 (1.4) |
| 22:4n6 | 0.3 (0.2)* | 0.1 (0.2) | 0.1 (0.1)ns | 0.1 (0.3) |
| 22:5n6 | 0.2 (0.3)ns | 0.1 (0.4) | 0.1 (0.4)ns | 0.2 (0.4) |
| 18:3n3 | 0.2 (0.3)ns | 0.1 (0.3) | 0.1 (0.3)ns | 0.2 (0.2) |
| 20:5n3 | 1.4 (1.1)* | 1.1 (0.8) | 1.1 (0.8)ns | 1.0 (0.7) |
| 22:5n3 | 1.0 (0.3)* | 0.5 (0.5) | 0.4 (0.5)ns | 0.6 (0.5) |
| 22:6n3 | 4.6 (1.9)* | 3.3 (1.8) | 3.4 (1.9)ns | 3.1 (1.6) |

*$P<0.001$ ($t$-test); †$P=0.006$ ($t$-test); ns – no significant difference (Mann–Whitney or $t$-test)

*Statistical analysis*

For the two groups of patients with bladder cancer, the means and standard deviations for each fatty acid were calculated. The two groups were then compared using the Mann–Whitney U-Test. The fatty acids levels in the two groups, both separately and in combination, were then compared with the normal controls using the unpaired $t$-test.

### Results

Of the 98 patients entered in the study, 55 had active disease (i.e. recurrence tumour at cystoscopy) and 43 had inactive disease. The age and sex distribution of the two groups is outlined in Table I, and shows that the two groups are comparable by age and sex.

The results of the fatty acid analysis of the plasma phospholipids of the two groups and a control population is shown in Table II. Comparison of all the cancer patients with the control population showed significantly lower plasma levels of the major dietary n-6 EFA, linoleic acid ($P<0.001$, $t$-test), and of all its metabolites except 20:3n6 (dihomogammalinolenic acid) and 22:5n6. The level of the major dietary n-3 EFA, alpha-linolenic acid, was not significantly lower than the control level, but the levels of its metabolites were significantly lower ($P<0.001$, $t$-test).

No significant differences were found, using the Mann–Whitney U-Test, between the active and inactive disease groups.

### Discussion

We have demonstrated significant differences in the plasma phospholipid profiles of patients with histologically proven bladder cancer, when compared to a control population. There were, however, no significant differences between those patients with active bladder disease and those with inactive disease. Measurement of the essential fatty acids in the plasma phospholipids of patients with bladder cancer is not, therefore, a useful marker of disease activity.

Changes in the plasma levels of the essential fatty acids in patients with cancer may be related to a pathological metabolism and incorporation into phospholipids of the various fatty acids. Similar variations have been demonstrated in patients with atopic eczema (Manku *et al.*, 1984), premenstrual syndrome (Brush *et al.*, 1984) and those at risk of developing coronary heart disease (Miettinen *et al.*, 1982).

It is possible that in patients with cancer, the cancer itself may be responsible for changes in essential fatty acid metabolism. Tumour cells tend to have a decreased activity of the desaturase enzymes, particularly the delta-6-desaturase (Reitz *et al.*, 1977; Bailey, 1977; Horrobin, 1980). This is in keeping with the known enzymatic differences between cancer cells and normal control cells, such as changes in enzymatic activity, concentration and composition (Weber, 1977; Pretlow *et al.*, 1985). This difference in activity of the tumour cell delta-6-desaturase leads to lower levels of the fatty acid derivatives in tumour cells. This seems to be mirrored in the plasma levels of the fatty acids, perhaps reflecting the diverse metabolic effects caused by the presence of a tumour. On the other hand, the lack of any difference between the active and inactive patients in our study may indicate that the presence of active tumour has no effect on systemic essential fatty acid metabolism.

An alternative equally interesting possibility is that the fatty acid abnormalities may in some way be involved in the pathogenesis of the tumour. A dietary deficiency of essential fatty acids does not reliably produce tumours except in the urinary tract. Rats deprived of dietary essential fatty acids consistently show pre-malignant and malignant change in the transitional epithelium of the urinary tract (Monis *et al.*, 1982). There is thus the possibility that a deficit in essential fatty acids, as we have shown in our patients, may predispose to the development of bladder cancer. It is even conceivable that essential fatty acid supplementation may be of value in preventing or treating bladder cancer.

### References

BAILEY, J.M. (1977). Cultured cells. In: Snyder, F. (ed.). *Lipid Metabolism in Mammals*. Vol II. Plenum Press: New York. pp. 323.

BRUSH, M.G., WATSON, S.J., HORROBIN, D.F. & MANKU, M.S. (1984). Abnormal essential fatty acid levels in plasma of women with premenstrual syndrome. *Am. J. Obstet. Gynecol.*, **10**, 363.

HORROBIN, D.F. (1980). The reversibility of cancer: the relevance of cyclic AMP, calcium, essential fatty acids and prostaglandin $E_1$. *Med. Hypotheses*, **6**, 469.

HORROBIN, D.F., ELLS, K., MORSE-FISHER, N. & MANKU, M.S. (1989). Fatty acid distribution in plasma phospholipids in normal individuals from different geographical locations. Presented at: *Am. Oil. Chem. Soc. Meeting*, Cincinatti, May 1989.

VAN HOUWELINGEN, A.C., KESTER, A.D.M., KROMHOUT, D. & HORNSTRA, G. (1989). Comparison between habitual intake of polyunsaturated fatty acids and their concentrations in serum lipid fractions. *Eur. J. Clin. Nutr.*, **43**, 11.

MANKU, M.S., HORROBIN, D.F., MORSE, N.L., WRIGHT, S. & BURTON, J.L. (1984). Essential fatty acids in the plasma phospholipids of patients with atopic eczema. *Br. J. Dermatol.*, **110**, 643.

MIETTINEN, T.A., NAUKKARINIEN, V., HUTTUNEN, J.K., MATTILA, S. & KUMLIN, T. (1982). Fatty acid composition of serum predicts myocardial infarction. *Br. Med. J.*, **285**, 993.

MONIS, B. & EYNARD, A.R. (1982). Abnormal cell proliferation and differentiation and urothelial tumorigenesis in essential fatty acid deficient rats. *Progr. Lipid Res.*, **20**, 691.

PRETLOW, T.G.II, HARRIS, B.E., BRADLEY, E.L.Jr., BUESCHEN, A.J., LLOYD, K.L. & PRETLOW, T.P. (1985). Enzyme activities in prostatic carcinoma related to Gleason grades. *Cancer Res.*, **45**, 442.

REITZ, R.C., THOMPSON, J.A. & MORRIS, H.P. (1977). Mitochondrial and microsomal phospholipids of Morris hepatoma 7777. *Cancer Res.*, **37**, 561.

WEBER, G. (1977). Enzymology of cancer cells. *N. Engl J. Med.*, **296**, 486.